# Post-traumatic Stress Disorder: A Major Source of Concern: Comment on "Epidemiology of Post-traumatic Stress Disorder in Iranian Population From 2019 to 2024: A Systematic Review and Meta-analysis"

**DOI:** 10.34172/aim.33448

**Published:** 2025-02-01

**Authors:** Vitorino Modesto Santos, Andressa Plaça Tedeschi, Laura Campos Modesto

**Affiliations:** ^1^Armed Forces Hospital, Catholic University of Brasília-DF, Brazil; ^2^Department of Medicine, University of the Americas, São Paulo-SP, Brazil; ^3^Department of Medicine, University Center (UNICEUB) of Brasília-DF, Brazil

## Introduction

 We read the timely and very illustrative review published in this journal by Imani et al focusing on the major aspects of the post-traumatic stress disorder (PTSD) in Iran.^1^ The data from 15 articles and a total of 9 868 people with a mean age of 35.61 ± 7.8 years showed a PTSD prevalence of 31.87%, including 36.64% in men and 35.52% in women. The study included cases registered between 2019 and 2024, and PTSD was associated with job-related traumas, accidents, wars, floods, COVID-19 infections, and childbirths.^1^ The authors emphasized the relatively high level of PTSD in Iran, the relationship with the occurrence of earthquakes, floods, storms, and road traffic accidents, besides the war. They also stressed the lack of a significant decrease in PTSD prevalence from 2019-2024 and suggested future studies on PTSD prevalence and the trend of its changes over time.^1^ In this setting, the aim of short additional comments on novel literature from 2024 is to highlight the first article of reference and increase the awareness of healthcare workers.

 Khoshakhlagh et al performed a cross-sectional study in 2022 about job stress, post-traumatic stress, and musculoskeletal symptoms; besides the role of job burnout and depression mediators, based on questionnaires to 2339 Iranian firefighters.^2^ The data showed that both high job stress and PTSD enhanced the chance (34%) of musculoskeletal symptoms; and if associated, they increased this probability by 37%. Depression was related to musculoskeletal symptoms more than burnout, while job stress and PTSD increased musculoskeletal symptoms influenced by burnout or depression.^2^ The authors concluded that preventive measures and the accurate control of burnout and depression mediators are critical for both the firefighter’s mental and physical health.^2^ Maddah et al evaluated, by interviews between June 2022 and January 2023, the challenges among 15 spouses of Iranian veterans who had been diagnosed with PTSD.^3^ The spouses’ mean age was 56.74 ± 6.43 years, and analysis of their responses revealed 7 categories including burnout, apathy towards self-care, depression, crushed and ignored, relationship disturbances, financial burden, besides declined social status.^3^ The data showed that PTSD in veterans directly and indirectly affects the living conditions of their spouses, causing emotional detachment, constant rejection, and resilience against stressors, being disruptive issues of their physical and mental health. The authors emphasized that those spouses also require social and therapeutic support.^3^ Masoudnia and Rahmati Farmani reviewed PTSD consequence data among war-torn immigrants with 45-years-old and over (121 women and 124 men) who migrated during the Iran-Iraq war.^4^ The prevalence of PTSD was 35.1%, and there was a significant negative correlation between perceived social support and PTSD, whereas a significant positive correlation was observed between both the avoidance coping strategies and self-control and PTSD. The authors emphasized social and behavioral interventions to enhance the support and strengthen individual control among the war-torn to minimize the development of PTSD.^4^ Pirzad Jahromi et al studied the steady-state evoked potential (SSEP) in 25 adults with PTSD and 25 healthy controls submitted to electroencephalography while the tone signal stimuli at 40 Hz were utilized to evoke SSEPs and subjects performed a stop-signal task.^5^ The patients showed poorer performance in the cognitive tasks, with raised SSEP phase and amplitude in the anterior and midline regions in comparison to the controls.^5^ The authors concluded that abnormalities observed in the anterior and midline cortical neural networks have important relationships with the pathophysiology of PTSD.^5^ Furthermore, these changes may constitute a clinically useful measure for researchers to evaluate biomarkers for early diagnosis of PTSD and new therapies and management.^5^ Omidvar Eshkalak et al performed a quasi-experimental study with intervention and control groups including 110 parents of children aged 10-18 years with PTSD diagnosis.^6^ The difference between the mean score of total traumatic stress and its subscales before intervention was not statistically significant; while after intervention, the scores decreased in the intervention group and increased in the control group with significant difference. The authors highlighted the role of parent training as a support for children with PTSD.^6^ Rahnejat et al compared prolonged exposure therapy (PET) with metacognitive therapy (MCT) in their effects on quality of life (QoL) among 57 veterans with PTSD.^7^ Veterans were distributed in 3 groups: MCT (n = 17), PET (n = 17), and Control (n = 23). The 36-item short-form survey evaluated QoL pre-test, post-test, and a 3-month follow-up. The MCT and PET groups had improved QoL at the post-test and follow-up, compared with the control group, but did not show a significant difference at the post-test or follow-up.^7^ The authors stressed the role of PET as a standard treatment for PTSD, in addition to the effectiveness of MCT at increasing the QoL in war-related PTSD during the follow-up.^7^ Rajabzadeh et al conducted a quasi-experimental study among parents of 80 preterm infants admitted to hospital in 2020 and categorized them as 40 in the intervention group and 40 controls.^8^ The intervention group had five daily family-centered sessions in the presence of both couples and for each couple separately, whereas the controls had only training and care.^8^ As the mean score changes in the intervention group were significantly higher than those in the controls, the authors concluded that a family-centered education has a positive effect on reducing the severity of posttraumatic stress in mothers of premature infants.^8^ Rashidi et al performed a randomized controlled trial to evaluate the role of nurse diary intervention on PTSD and recall of memories in ICU survivors, including 30 patients from trauma intensive care unities distributed as control and intervention groups.^9^ The total mean PTSD score in the intervention group was lower than that among controls; those in the intervention group who remembered their admission, the hospital stay before the ICU care, and all their ICU stay, were more numerous than those of the control group.^9^ The authors concluded that the nurse-initiated diary is effective on PTSD and recall clear memories of patients admitted to the ICU, and suggested the use of this intervention.^9^

 Additional literature comments emphasized the cornerstone first reference.
